# Effect of Physical Exercise in Real-World Settings on Executive Function of Typical Children and Adolescents: A Systematic Review

**DOI:** 10.3390/brainsci12121734

**Published:** 2022-12-18

**Authors:** Peng Shi, Yan Tang, Ziyun Zhang, Xiaosu Feng, Chenyang Li

**Affiliations:** 1 School of Physical Education, Shanghai University of Sport, Shanghai 200438, China; 2 School of Physical Education, Liaoning Normal University, Dalian 116029, China

**Keywords:** motor skills, executive function, children and adolescents, typical development, physical exercise, real-world settings

## Abstract

Objective: The aim of this paper is to provide a systematic review of research on physical exercise in real-world settings on executive function of typical children and adolescents. Methods: The CNKI, WOS, PubMed, ScienceDirect, and SPORTDiscus databases were searched by computer. Two researchers independently screened the literature, extracted data, and evaluated the risk of bias in the included literature. Statistical analysis was performed using frequency and percentage and the *χ*^2^ test. Results: A total of 49 articles was included. Acute (moderate intensity lasting 30–50 min) and long-term (interventions of moderate intensity of 30–50 min at least 3 times a week for 17 weeks or more) physical exercises in real-world settings have positive intervention effects on executive function. Furthermore, for acute interventions, closed skills are more efficient for inhibitory control, open skills are more efficient for working memory and cognitive flexibility, and open-continuous and closed-sequential skills are the most efficient; long-term interventions with open skills, sequential skills, and open-sequential skills are more effective. Conclusion: Physical exercise in real-world settings has a good promotion effect on typical children and adolescents, and motor skills with open and/or sequential attributes are more helpful in improving executive function.

## 1. Introduction

Executive function can be described as a higher-level, top-down thinking process that is closely related to frontal brain activity [1]. It is the process by which individuals coordinate divergent cognitive activities while undertaking a cognitive task, with the objective of enabling individuals to achieve set targets and produce intentional behavior in a flexible and efficient way [2]. In general, the executive function is recognized as a multidimensional structure [3]. Although there is an ongoing debate over the elements of executive function, the general consensus is that executive function includes flexibility, goal-setting and planning, attention and memory systems (such as working memory), and inhibitory control [4,5]. Children and adolescents are at a peak cognitive-development stage. The level of executive-function development during this period is crucial for academic achievement, physical and mental health, and social adaptation [6,7,8]. As a corollary, it is crucial to study the cognitive development and facilitation strategies of children and adolescents for the benefit of their long-term psychological and physiological health.

In sports-science research, the benefits of physical exercise on the physical and mental health of children and adolescents have been a central focus. An increasing number of studies [9,10,11] confirm the positive effects of physical exercise on brain growth and cognitive development in children and adolescents. Physical exercise increases the plasticity of gray- and white-matter structures [12,13], enhances the state of brain activation during specific tasks [14], and strengthens functional brain networks [15], thereby bolstering executive function in children and adolescents. Early research on the effects of exercise interventions on the executive function of children and adolescents was conducted primarily in laboratory settings. Their interventions, which primarily involved treadmills and power bikes, disregarded the complexity of movement in real-world settings and lacked ecological validity [16]. Moreover, positive laboratory results indicate that running and cycling are efficient methods for enhancing executive function. Running and cycling can be monotonous for children and adolescents, who are more likely to engage in other forms of physical activity [17]. The advantage of real-world settings is that equipment requirements are less complicated and easier to incorporate into in- or extra-curricular physical activities [18]. Therefore, Vazou et al. [19] called on researchers to conduct more real-world studies to determine which type of exercise intervention is the most effective. Pesce et al. [20] and Diamond et al. [21] believe that the restriction of focusing solely on quantitative characteristics of sports (intensity, period, frequency, and duration, etc.) should be broken and attention should be shifted to the qualitative aspects of sports (metabolic function, motor skill type, etc.).

Physical exercise is based on motor skills. Motor skills are operational activities that are acquired through learning. Based on the predictability of the surrounding environment, motor skills can be categorized into open and closed skills [22]. Open skills refer to the activities of performing motor tasks in an unpredictably changing environment, requiring individuals to react and adapt their movements. Closed skills refer to motor tasks performed in a stable and predictable environment, where individuals can plan their motor routines in advance [22]. Therefore, there may be a difference in the efficacy of interventions for executive function between open and closed skills. A recent meta-analysis [23] examined the effects of open and closed skills on cognitive function in children, adults, and the elderly and reported that open skills enhanced cognitive function more than closed skills. The dynamic interaction between “individual–environment–task,” in which individuals are required to engage in more cognitive and decision-making processes, strengthens brain structure and function by coordinating and consolidating existing movements and creating novel movements [24,25].

Nevertheless, a number of studies [26,27] have uncovered no distinction between the effects of open and closed skills on executive function. This could be due to the fact that the structure of the movement may modulate the effects of open- and closed-skill interventions on executive functions. The number of joints involved in a movement reflects the complexity of the structure of the movement; the more joints involved in the movement, the more body coordination is facilitated [28]. Multi-articular, cognitively demanding motor-repetition exercises help to activate brain-related neural circuitry [24]. Numerous studies [29,30] have also shown a strong link between motor coordination and executive function in children and adolescents; impaired executive function is the central deficit in developmental coordination disorder (DCD) [31].

In addition, motor skills cannot effectively distinguish between activity tasks through a single dimension. For instance, the motor structure of aerobics and middle-distance running, which are both closed skills, differs significantly. Basketball and Tai Chi, which are both sequence skills, have distinct environmental contexts and cognitive-participation differences. Motor skills can be classified as sequential or continuous, depending on the complexity of the movement structure [22]. Sequential skills are more complex motor sequences that link multiple discrete motors in a particular order. Continuous skills are multiple repetitions of a single discrete motor without a peculiar beginning or end and a relatively uniform motor structure [22]. The classification systems of Schmidt et al. [32] and Voss et al. [33] were applied to develop four types of motor skills for this study: open–sequential skills, open–continuous skills, closed–sequential skills, and closed–continuous skills.

Consequently, this study has the following three research objectives: (1) to systematically review the effects of real-world exercise on the executive function of typical children and adolescents; (2) to explore the moderating effect of quantitative characteristics of interventions on executive function; and (3) to investigate the moderating effect of motor skill types on executive function.

## 2. Materials and Methods

This review was registered (CRD42022348781) in the International Prospective Register of Systematic Reviews (PROSPERO). The Preferred Reporting Items for Systematic Reviews and Meta-Analyses (PRISMA 2020) guidelines [21] were followed for this study.

### 2.1. Search Strategy

An individual researcher used keywords to search the relevant literature. The China National Knowledge Infrastructure (CNKI), Web of Science (WOS), PubMed, ScienceDirect, and SPORTDiscus databases were scrutinized for pertinent literature. In addition, Google Scholar searches were employed to identify literature that may have been overlooked. The search date ranges from the creation of the database to May 2022. In this study, a combination search with the following three sets of subject terms was conducted: (1) motor skill OR sports skill OR sports items OR sports types OR exercise; (2) executive function OR working memory OR inhibition control OR inhibitory control OR cognitive flexibility OR self-control; and (3) children OR child OR adolescent OR teenagers OR young. The Boolean logical operator “AND” is used to join three groups of subject terms. Similar search terms were applied to search the titles and keywords of the databases listed above. In addition, references in the obtained articles were reviewed.

### 2.2. Selection Criteria

According to PICOS principles [34], inclusion and exclusion criteria for the literature were formulated. The inclusion criteria were as follows: (1) subjects were typical children and adolescents; (2) exercise interventions in real-world settings were acute and long-term; (3) control measures included traditional physical-education courses, basic academic courses, free activities, or being seated; (4) outcome variables consisted of planning, inhibitory control, working memory, and cognitive flexibility; and (5) study designs included a randomized controlled trial (RCT), randomized crossover design (RCD), non-randomized concurrent control trial (non-RCCT), and before–after study (BAS). The exclusion criteria were as follows: (1) cross-sectional, case-control, and other descriptive studies; (2) reviews, abstracts, letters, and comments lacking a clear description of the study design; (3) screen-based physical games, such as Xbox and Kinect; (4) combined physical exercise and cognitive-therapy interventions; and (5) in the case of duplicate publications, only the superior-quality literature was considered for inclusion. The order of title, abstract, figure, and full text determined the literature-selection procedure. The selection of the literature was carried out independently by two researchers each, with two other researchers conducting a secondary assessment of the selected literature. In the event of a disagreement between the two groups, all researchers would exchange assessments and reach a consensus.

### 2.3. Data Extraction

First author, publication date, study design, subject characteristics (sample size, age, and percentage of females), interventions (sports items, intervention period, weekly frequency, session duration, and intensity), control measures, and outcome variables (measures and results) were entered into Excel 2010 and stored. In addition, sports activities were categorized into a classification system for motor skills. The data extraction was undertaken independently by two researchers, and the extracted data were reviewed by two additional researchers. In the event of a disagreement between the two groups, all researchers exchanged assessments and reached a consensus.

### 2.4. Quality Assessment

The quality of RCTs and RCDs was evaluated utilizing the risk-of-bias assessment tool recommended by the Cochrane Collaboration Network [35]. Six aspects of the tool were evaluated: randomization methods, blinding, allocation concealment, completeness of outcome data, selective reporting of study results, and other biases. The methodological index for non-randomized studies (MINORS) [36] scale was utilized to assess the quality of non-RCCTs and BAS. The tool consists of 12 entries, of which 9 to 12 are additional criteria used to evaluate studies with a control group. Each entry is assigned a score of 2, for a total score of 24. A score of 0 indicates “not reported”, 1 means “reported with insufficient information”, and 2 denotes “reported with sufficient information”. Two researchers independently evaluated the assessment tools. In case of significant disagreements, they were discussed with a third researcher.

### 2.5. Statistical Methods

Due to the differences in research paradigms and measurement tools in the included literature, it was difficult to estimate effect sizes using meta-analysis; therefore, this study only employed a systematic-review approach to evaluate research results in this area. For statistical analysis, the Statistical Product Service Solutions (SPSS) 25.0 software (developed by IBM of New York State, New York, NY, USA) was utilized. Utilizing frequencies and percentages, descriptive statistics on the number of articles with efficient interventions were compiled. The *χ*^2^ test was utilized to compare between-group differences in the efficacy of the intervention.

## 3. Results

### 3.1. Literature-Selection Results

**Figure 1:**
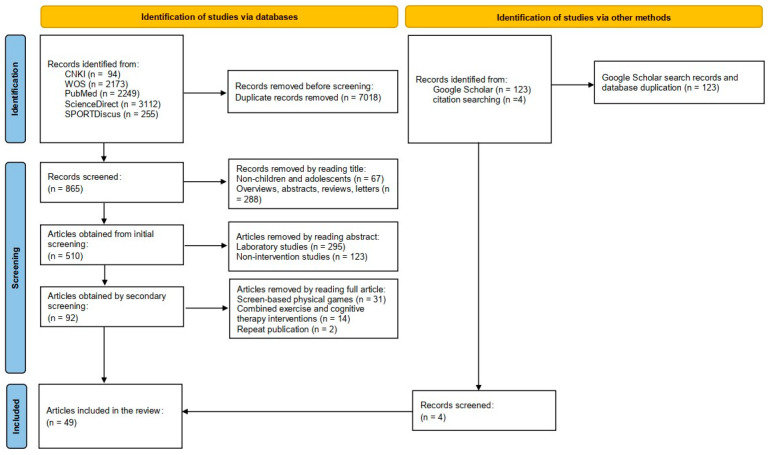
Literature-selection process according to the PRISMA 2020 guideline.

The search strategy retrieved a total yield of 8010 articles. The retrieved articles were imported into EndNote X9 software for de-duplication, and 869 articles were obtained. After further selection of the articles, a total of 49 articles was finally included. Figure 1 illustrates the study-selection process according to the PRISMA 2020 guideline.

### 3.2. Data-Extraction Results

A total of 49 articles that satisfied the criteria for inclusion in the systematic review were accepted; among those 49 articles, 14 (28.6%) articles discuss acute interventions for typical children and adolescents and 35 (71.4%) articles delve into long-term interventions for typical children and adolescents. A total of 38 (77.6%) of the included studies were RCTs, 2 (4.1%) were RCDs, 8 (16.3%) were non-RCTs, and 1 (2.0%) was a BAS. There were 6079 children and adolescents aged 3 to 18 included in the 65 articles. A total of 34 (69.4%) articles reported the proportion of female subjects, with two [37,38] interventions and one [39] intervention focusing on boys and girls only, whereas the remainder utilized the entire sample, with the proportion of females ranging from 18.8% to 71.0%. The acute intervention lasted between 10 and 50 min. Ten articles (71.4%) reported the intensity of exercise, and the majority (70%) were moderate. The quantitative features of the long-term intervention were 4~36 weeks, 1~7 times/week, and 20~120 min/time. The majority (77.3%) of the 22 articles that addressed the intensity of the exercise were of moderate intensity. Six (12.3%) of the articles mentioned planning, 37 (75.5%) acknowledged inhibitory control, 31 (63.3%) reported working memory, and 21 (42.9%) discussed cognitive flexibility. In addition, the literature on acute interventions included seven [37,40,41,42,43,44,45] multi-arm studies for a total of 22 studies, whereas the literature on long-term interventions contained 13 [46,47,48,49,50,51,52,53,54,55] multi-arm studies, for a total of 48 studies. The characteristics of the included studies are detailed in Table 1.

### 3.3. Quality-Assessment Results

Randomization procedures were described in 17 (42.5% of all articles) of the 40 randomized trials (RCTs and RCDs), the majority of which involved block randomization, cluster randomization, and stratified randomization. Sixteen articles (40%) reported blinding strategies, primarily subject blinding, assessor blinding, and double blinding. Only one [48] reported an allocation-concealment strategy. In 25 (62.5%) of the articles, complete outcome data were reported. There was no selective reporting of study results in any of the articles, and the existence of other biases was unidentified (Table 2). The quality scores for the nine non-randomized trials (non-RCCTs and BAS) ranged from 17 to 24; the quality score for BAS was 13, indicating relatively high-quality included literature. The primary reasons for the lower quality score of the included literature were that endpoint-indicator evaluation was not conducted using blinded methods, the sample size was not estimated, and the proportion of lost follow-up was >5% (Table 3).

### 3.4. Results of the Systematic Review

#### 3.4.1. Acute-Intervention Effects

In the 18, 16, and 13 studies that examined the effects of acute exercise interventions on inhibitory control, working memory, and cognitive flexibility in typical children and adolescents, respectively, 14 (77.8%), 12 (75.0%), and 7 (53.8%) of those studies, respectively, revealed beneficial effects following the intervention. Inhibitory control and working memory were significantly more responsive to acute interventions than cognitive flexibility. The efficiency of the intervention was not statistically significant between these three indicators (*χ*^2^ = 2.333, *p* = 0.331) (Table 4). In terms of inhibitory control and cognitive flexibility, there was no significant difference between pre-school and post-preschool children (*p* > 0.05). Nonetheless, the literature on acute interventions for preschool-aged children is scant [58,62], necessitating further research. There were no significant differences in efficiency between intensities (*p* > 0.05). However, the moderate intensity was more than 75% in terms of inhibitory control, working memory, and cognitive flexibility. In addition, working memory (*χ*^2^ = 6.857, *p* < 0.01) and cognitive flexibility (*χ*^2^ = 4.550, *p* < 0.05) showed significant differences between the intervention efficiency at 30–50 min per session compared to 10–20 min per session. Closed skills demonstrated significantly greater intervention efficiency on inhibitory control than open skills (*χ*^2^ = 4.018, *p* < 0.05). However, the intervention efficiency of closed skills in terms of working memory and cognitive flexibility was marginally inferior to that of open skills. Between continuous and sequential skills, there was no discernible difference in executive function (*p* > 0.05). There was a significant difference in the efficient intervention rate for inhibitory control by various types of motor skills under the two-dimensional skill-type system (*χ*^2^ = 8.839, *p* < 0.05); among which the most efficient intervention rates were exhibited by open–continuous and closed–sequential skills. The overall efficient intervention rate was highest for open–continuous skills, followed by closed–sequential skills.

#### 3.4.2. Long-Term Intervention Effects

Of the 7, 36, 31, and 20 studies that examined the effects of long-term exercise interventions on planning, inhibitory control, working memory, and cognitive flexibility, respectively, 4 (57.1%), 25 (69.4%), 19 (61.3%), and 12 (60.0%) studies, respectively, revealed beneficial effects following the intervention. Long-term interventions were slightly more effective on inhibitory control and working memory compared to cognitive flexibility and planning. The efficiency of the intervention was not statistically significant between these four indicators (*χ*^2^ = 0.841, *p* = 0.840) (Table 5). There was no significant difference between pre-school and post-preschool children (*p* > 0.05). However, the literature on long-term interventions for preschool-aged children is scant [47,65,70,74,80,83], thus necessitating additional research. The efficiency of different intensities of intervention on the executive function of children and adolescents did not differ significantly (*p* > 0.05). The efficiency of moderate-intensity interventions on inhibitory control, working memory, and cognitive flexibility exceeded 75%. In terms of intervention efficiency, there were no significant differences between acute and long-term interventions for inhibitory control (*χ*^2^ = 0.415), working memory (*χ*^2^ = 0.883), and cognitive flexibility (*χ*^2^ = 0.122) in typical children and adolescents (*p* > 0.05). Moreover, there were no statistically significant differences in executive function in terms of the intervention period (*p* > 0.05). At 17 weeks or more, however, the intervention was more efficient for inhibitory control and working memory. There were no significant differences in intervention efficiency between different intervention frequencies for executive function (*p* > 0.05). Although the efficiency of ≥3 x/week in inhibitory control, working memory, and cognitive flexibility was over 70%, the efficiency of different durations on the executive function of typical children and adolescents was not statistically significant (*p* > 0.05). However, interventions lasting 30–50 min were more efficient on average. In terms of skill type, open skills were more efficient at intervening in executive function than closed skills, particularly in the dimension of inhibitory control (*χ*^2^ = 5.740, *p* < 0.05). Though there was no statistically significant difference between continuous and sequential skills in terms of the efficiency of executive-function interventions (*p* > 0.05), sequential skills were more efficient overall. Under the two-dimensional skill-type system, there were significant differences in the intervention efficiency of different types of motor skills on inhibitory control (*χ*^2^ = 9.555, *p* < 0.05). In general, open–sequential skills were the most efficient form of intervention.

## 4. Discussion

### 4.1. Overall Effect of Physical Exercise in Real-World Settings

In typical children and adolescents, acute and long-term physical exercise in real-world settings was more than 50% efficient in all aspects of executive function and more efficient in inhibitory control and working memory than in cognitive flexibility and planning. Several researchers [86,87] differentiated executive function into three substructures: inhibitory control, working memory, and cognitive flexibility. However, according to Smith and Jonides [88], executive function extends far beyond these low-level structures and should include highly relevant real-world components such as task management, goal planning, monitoring, and coding. Compared to inhibitory control and working memory, cognitive flexibility is a more complex executive function based on the development and coordination of inhibitory control and working memory [89]. Likewise, planning and problem-solving require the collaborative processing of numerous higher-order cognitive processes. For instance, the Rey Complex Figure Test (RCFT) and tower tasks (e.g., the Tower of Hanoi and the Tower of London) are typically controlled by goal orientation, planning and organization, maintaining goals in working memory, and monitoring behavioral performance [90,91]. Consequently, advancements in the higher structures of executive function are dependent on lower structures. Further improvements in cognitive flexibility and planning are only possible through the efficient development of inhibitory control and working memory. Several studies [41,50,51,54,55,61,62,65,66,70,75] also suggest that the effects of exercise interventions on cognitive flexibility and planning are insignificant in the absence of efficient improvements in inhibitory control or working memory.

### 4.2. Demographic Variables

There were no significant differences in the effectiveness of the intervention between the pre-school and post-preschool groups. This result contradicts the findings of a prior study on cognitive training, which concluded that younger children had greater potential for training-induced improvement and demonstrated greater training benefits on cognitive tasks [18]. However, executive function is accompanied by staged maturation and development of the prefrontal lobe, with the fastest-developing stages occurring between 0 and 2 years of age, 7 and 9 years of age, and 16 and 19 years of age [92]. Considering the lengthy maturation period of the prefrontal cortex, one study [89] revealed that executive functions may not be fully developed until after the age of 20. According to the findings of a recent meta-analysis, age factors did not have any influence on the facilitative effect of physical activity on executive function [93]. Consequently, the intervention was equally efficient with older adolescent age groups.

Only three articles [37,38,39] involved interventions with children of a single gender, and the complexity and inconsistency of the interventions made it difficult to draw conclusions about gender. Based on the findings of one study [94], short bursts of aerobic exercise at varying intensities exhibited selectively beneficial effects on the executive function that did not differ by gender. However, girls typically display a greater level of executive function and may have less room for growth than boys [18]. In addition, boys are more likely to engage in more intense open-ended activities, which may be more advantageous in terms of executive function. Nevertheless, no comparative studies on the effects of real-world environmental exercise on executive function in different age and gender groups were retrieved.

### 4.3. Quantitative Characteristics of the Intervention

Acute or long-term interventions of moderate intensity were generally more efficient than those of low or high intensity, similar to the findings of previous studies [41,47] evaluating dose–effect relationships. In accordance with the model of self-control strength, self-control energy is constrained. If the energy expended by previous self-control cannot be recovered in a timely manner, it may result in self-depletion, which will affect future self-control [95]. This study lends support to the self-control strength model, which posits a U-shaped relationship between intervention intensity and executive function. The intervention over 17 weeks has the highest overall intervention efficiency. Short-term interventions can bolster levels of activation in the dorsolateral prefrontal cortex and boost blood flow to the brain, according to neuroimaging studies [11]. Sustained interventions can enhance the structural plasticity of the brain’s gray and white matter and augment the functionality of its neural networks [10]. Consequently, the latter features a greater intervention effect [12]. In terms of frequency, interventions that occur more frequently than three times per week have a greater impact on executive function overall. A linear dose–effect relationship between exercise frequency and cognitive benefits was discovered [96,97]; that is, the greater the frequency of exercise, the greater the cognitive benefits. Acute interventions lasting 30–50 min were more efficient than those lasting 10–20 min. Interventions lasting 30–50 min were more efficient than those lasting less than 30 min or longer than 50 min. This result is in line with previous research [60,98]. A possible explanation is that the duration of the intervention has an inverted U-shaped dose–effect relationship with executive function, with both longer and shorter interventions likely to diminish cognitive benefits [98]. Specifically, sustained interventions can deplete resources for self-control and result in a decline in cognitive tasks [99].

### 4.4. Types of Skills of the Intervention

The improvement of executive function benefits from the qualitative characteristics of exercise interventions. First, closed skills were more efficient in acute interventions for inhibitory control and open skills for working memory and cognitive flexibility, whereas open skills were more efficient in long-term interventions. The differential effects of various types of motor skills on brain tissue and neural activation may account for the selective enhancement of executive function by acute interventions. Closed skills, such as jogging, cycling, and aerobics, improve a person’s cardiorespiratory fitness, increase the capillary density of brain tissue, and activate the sensorimotor network involved in the regulation of response inhibition [100]. Open skills such as basketball and table tennis stimulate individual perceptual–motor coordination, expand the amount of Purkinje neurons and synapses, and activate the visuospatial network involved in attention control and working memory [100,101]. Long-term interventions in open skills necessitate not only an abundance of environmental stimulation but also an emphasis on cardiorespiratory fitness [102,103]. They were able to effectively activate sensorimotor and visuospatial networks, induce better neurological remodeling, and lead to greater improvements in executive function.

Second, sequential and continuous skills were equally efficient in the acute intervention, whereas sequential skills were generally more efficient in the long-term intervention. Sequential skills include a more complex movement structure, and the multi-limb involvement in the task necessitates more mental-manipulation processes [102]. This makes it easier to improve executive function than with purely continuous activities. A neuroimaging study [104] also revealed that complex random movements induce neurogenesis in the hippocampus, cerebellum, and cerebral cortex more readily than simple repetitive movements.

Finally, this study discovered that open–continuous and closed–sequential skills were the most efficient interventions in acute intervention, premised on a two-dimensional classification system for motor skills. In the long-term intervention, open-sequential skills were the most efficient interventions. The outcome is similar to the findings of the comparison of the one-dimensional skill-type system. To summarize, not all forms of physical exercise offer the same advantages for executive function [15,105]. Most studies [37,42,50,51,53] indicated that complex motor skills with open and/or sequential characteristics are more beneficial for enhancing executive function in children and adolescents. Krafft et al. [106] also evidenced a favorable intervention effect of aerobics (closed–sequential) on executive function rated by teachers. Based on this result, we should design intervention programs based on selective facilitation of executive function by different types of motor skills. For intervention, it is recommended to combine skills with a diversified and cognitively challenging environment.

### 4.5. Limitations

First, there is a possibility of bias in the selection of studies, which can compromise the reliability of the findings. Second, despite the overwhelming interest in the effect sizes of exercise interventions, it is a daunting task to test for combined effects due to the high heterogeneity in the evaluation criteria (response time, accuracy, and score) of the measurement instruments used in the studies. Finally, the limitations of primary data have prevented researchers of this paper from investigating the time-course effects of motor-skill interventions on executive function in children and adolescents; hence, it is undetermined how long the cognitive benefits generated by the interventions may be sustained.

## 5. Conclusions

This systematic review demonstrates the beneficial effects of physical exercise on executive function in children and adolescents, both short-term and long-term, in real-world settings. Exercise interventions exhibit comparable effects in pre-school and post-preschool age groups. The more efficient acute interventions are moderately intense and last 30–50 min. Interventions of moderate intensity that last 30–50 min at least three times a week for 17 weeks or more are more effective. In addition, for acute interventions, closed skills are more efficient for inhibitory control, open skills are more efficient for working memory and cognitive flexibility, continuous and sequential skills were similarly efficient, and open–continuous and closed–sequential skills are the most efficient. Long-term interventions with open skills, sequential skills, and open-sequential skills are more effective. The above results provide a basis for the development of well-designed interventions that can contribute to effective practice in school exercise settings.

## Figures and Tables

**Table 1:** Characteristics of included studies.

Included Article Study Design	Patients (N/Age/F%)	Interventions and Controls	Outcome	
			Outcome Measures	Results
Budde et al. [56], 2008 RCT	E = 47/15.04 ± 0.87y/22.9% C = 52/15.04 ± 0.87y/15.4%	10 min moderate-intensity soccer exercise (O, S) (E) vs. general activity (C)	②d2-test	+
Niemann et al. [57], 2013 RCT	E = 27/9.7 ± 0.4y/NC C = 15/9.7 ± 0.5y/NC	12 min high-intensity (85–90% HRmax) track-and-field run (Cl, Co) (E) vs. sitting (C)	②d2-test	+
Palmer et al. [58], 2013 RCD	16/49.4 ± 5.3 m/18.8%	30 min of passing, dribbling, and throwing activities (O, S) (E) vs. sitting (C)	②PDTP	0
Yan et al. [40], 2014 RCT	E1 = 52/9.8 ± 0.3y/53.8% E2 = 51/9.7 ± 0.3y/49.0% C = 51/9.8 ± 0.3y/49.0%	30 min moderate-intensity (60–69% HRmax) aerobics (Cl, S) (E1) vs. obstacle run (O, Co) (E2) vs. sitting (C)	②Flanker (E1 > E2) ③1-back (E1 > E2) ④More-odd shifting (E2 > E1)	+& +& +&
Chen et al. [41], 2014a RCT	E1 = 30/9.8 ± 0.3y/50.0% E2 = 30/9.8 ± 0.3y/53.3% E3 = 32/9.7 ± 0.3y/46.9% C = 28/9.8 ± 0.3y/50.0%	30 min low-intensity (50–59% HRmax) basketball high dribbling and dribbling between runs (Cl, S) (E1) vs. moderate intensity (60–69% HRmax) (E2) vs. high intensity (70–79% HRmax) (E3) vs. free activities in their classroom (C)	②Flanker (E2 > E1 = E3 > C) ③1-back (E2 = E3 > E1 = C) ④More-odd shifting (E2 > E3 = C > E1)	+& +0& +−0&
Chen et al. [59], 2014b RCT	E = 44/3~5g/47.7% C = 38/3~5g/55.3%	30 min moderate-intensity (60–70% HRmax) track-and-field run (Cl, Co) (E) vs. sedentary reading (C)	②Flanker ③2-back ④More-odd shifting	+ + +
Chen et al. [42], 2015a RCT	E1 = 39/9.1 ± 0.3y/48.7% E2 = 38/9.1 ± 0.3y/44.7% C = 38/9.2 ± 0.4y/77.7%	30 min moderate-intensity (60–69% HRmax) cooperative rope skipping (O, Co) (E1) vs. single rope skipping (Cl, Co) (E2) vs. sedentary reading (C)	②Flanker (E1 > E2) ③1-back (E1 > E2) ④More-odd shifting (E1 > E2)	+& +& +&
Chen et al. [60], 2015b RCT	E = 24/9.5 ± 0.3y/NC C = 22/9.5 ± 0.3y/NC	30 min moderate-intensity (60–69% HRmax) basketball high dribbling and dribbling between runs (Cl, S) (E) vs. free activities in their classroom (C)	②Flanker (E1 > C) ③1-back (E1 > C) ④More-odd shifting (E1 > C)	+ + +
Jäger et al. [43], 2015 RCT	E1 = 54/134.6 ± 6.6 m/64.8% E2 = 62/135.3 ± 6.5 m/45.2% C = 58/135.8 ± 6.3 m/56.9%	20 min moderate-intensity (70% HRmax) cognitive-involvement skill games (O, S) (E1) vs. aerobic exercise without cognitive involvement (Cl, Co) (E2) vs. sitting without cognitive involvement (C)	②Flanker (E1 = E2) ③1-back (E1 = E2) ④More-odd shifting (E1 = E2)	0& 0& 0&
Gallotta et al. [44], 2015a RCT	E1 = 31/8~11y/NC E2 = 46/8~11y/NC C = 39/8~11y/NC	50 min traditional PE course (brisk walking, jogging, jumping, etc.) (Cl, Co) (E1) vs. basketball-skills acquisition practice (O, S) (E2) vs. basic academic course (C)	②d2-test (E1 > C > E2)	+−&
Cooper et al. [61], 2016 RCD	44/12.6 ± 0.6/52.3%	10 min high-intensity interval sprint in athletics hall (Cl, Co) (E) vs. sitting (C)	②Stroop ③Corsi blocks test ④DSST	+ 0 0
Stein et al. [62], 2017 RCT	E = 48/72.2 ± 5.2 m/50.0% C = 53/72.3 ± 6.9 m/52.8%	20 min motor-skill-learning practice based on coordination of both sides of the body (Cl, S) (E) vs. board game (C)	②Simon-says task ②Hearts and Flowers task-incongruent block ④Hearts and Flowers task-mixed block	+ 0 0
O’Brien et al. [37], 2021 RCT	E1 = 16/7.0 ± 0.5y/0.0% E2 = 16/6.7 ± 0.1y/0.0% C = 19/7.0 ± 0.5y/0.0%	30 min open-skills activities such as basketball, football, tennis (O, S) (E1) vs. closed-skills activities such as race, rope skipping, circuit training (Cl, Co) (E2) vs. free activities in their classroom (C)	③Backward Digit Span (E1 > E2) ③Corsi blocks test ③Motor span task (E2 > E1)	+& 0 +&
Ottoboni et al. [45], 2021 RCT	125/7~10y/NC	30 min high-intensity (170~180 bpm) team ball games (O, S) (E1) vs. agility obstacle run (O, Co) (E2) vs. basic academic course (C)	③Digit Span (E1 > E2) ③Corsi blocks test (E1 > E2)	+& +&
Manjunath et al. [39], 2001 RCT	E = 10/10~13y/100.0% C = 10/10~13y/100.0%	4 weeks (7 x/week) yoga intervention (Cl, S), 75 min/time (E) vs. traditional PE course (C)	①Tower of London	+
Lakes et al. [63], 2004 RCT	207/Kindergarten to Primary 5/NC	12 weeks (2–3 x/week) martial-arts intervention (Cl, S), 45 min/time (E) vs. traditional PE course (C)	③Digit Span	0
Davis et al. [46], 2011 RCT	E1 = 55/7~11y/NC E2 = 56/7~11y/NC C = 60/7~11y/NC	13 weeks (7 x/week) moderate-intensity (>150 bpm) running games, rope skipping, football and basketball exercise intervention, 20 min/time (E1) vs. 40 min/time (E2) vs. blank control (C)	①Cognitive Assessment System-Planning (E2 > E1 = C)	+0&
Kamijo et al. [64], 2011 RCT	E = 20/8.9 ± 0.5y/55.0% C = 16/9.1 ± 0.6y/50.0%	36 weeks (7 x/week) moderate- to high-intensity physical training combined with dribbling-skills practice, 70 min/time (E) vs. blank control (C)	③Sternberg	+
Fisher et al. [65], 2011 RCT	E = 34/6.1 ± 0.3y/53.0% C = 30/6.2 ± 0.3y/58.0%	10 weeks aerobic program, 1~2 h/week (E) vs. traditional PE course (C)	①Cognitive Assessment System-Planning ②Attention Network Test (ANT) ③Cambridge Neuropsychological Test Battery(CANTAB)-Spatial working memory	0 0 +
Chang et al. [47], 2013 non-RCCT	E1 = 13/7.2 ± 0.3/46.2% E2 = 13/7.0 ± 0.3/53.9%	8 weeks (2 w/week) low-intensity (40–50% HRmax) football learning practice (O, S), 2 sessions/week, 35 min/time (E1) vs. moderate intensity (60–70% HRmax) (E2) vs. pre-test	②Flanker (E1: Post > Pre; E2; Post > Pre)	+
Lakes et al. [66], 2013 RCT	E = 50/12.2y/52.00% C = 31/12.3y/48.00%	36 weeks (2 x/week) Taekwondo (Cl, S), 45 min/time (E) vs. traditional PE course (C)	②Hearts and Flowers task-incongruent block ④Hearts and Flowers task-mixed block	0 0
Telles et al. [48], 2013 RCT	E1 = 49/10.4 ± 1.2y/30.6% E2 = 49/10.5 ± 1.3y/46.9%	12 weeks (5 x/week) yoga (Cl, S), 45 min/time (E1) vs. physical exercise such as jogging, sprint running, relay races (Cl, Co) (E2) vs. pre-test	②Stroop (E1 < E2)	+&
Crova et al. [67], 2014 RCT	E = 37/9.6 ± 0.5y/46.0% C = 33/9.6 ± 0.5y/54.6%	21 weeks (1 x/week) moderate-intensity (150.5 ± 6.4 bpm) tennis (O, S), 120 min/time (E) vs. traditional PE course (C)	②RNG-inhibition of mental routines ③RNG-working memory updating	+ 0
Yin et al. [49], 2014 RCT	326/3~5 g/47.9%	20 weeks (3 x/week) moderate-intensity (120–140 bpm) martial arts + rope skipping+ 8 word run (Cl), 30 min/time (E1) vs. pattern running (O, Co), 5 x/week (E2) vs. blank control (C)	②Flanker (E1 > E2 = C) ③2-back (E2 > E1) ④More-odd shifting (E1 = E2)	+0& +& +&
Hillman et al. [68], 2014 RCT	E = 109/8.7~8.9y/49.0% C = 112/8.7~8.9y/44.0%	36 weeks (7 x/week) moderate-intensity (137.0 ± 68.3 bpm) aerobic exercise intervention, 120 min/time (E) vs. wait control (C)	②Flanker ③Switch task	+ +
Krafft et al. [69], 2014 RCT	E = 24/9.7 ± 0.8y/71% C = 19/9.9 ± 0.9y/58%	32 weeks (7 x/week) moderate intensity (161 ± 9 bpm) rope skipping and tag games, 40 min/time (E) vs. sedentary attention control (C)	②Flanker ②Antisaccade task	+ +
Yin et al. [50], 2015 RCT	610/3~5 g/46.9%	10 weeks (5 week) 40–80% HRmax pattern running (O, Co), 30 min/time (E1) vs. fun track-and-field games (O, Co), 3 x/week (E2) vs. small handball and physical-fitness exercises, 3 x/week (E3) vs. shuttlecock and games (E4) vs. martial arts, rope skipping, 8-word run (Co), 3 x/week (E5) vs. regular extracurricular physical activity (C)	②Flanker (E5 > E4 > E2 > E1 = E3 = C) ③2-back (E1 = E5 > E2 = E3 = E4 = C) ④More-odd shifting (E1 = E5 > E3 > E2 = E4 = C)	+0& +0& +0&
Jiang et al. [70], 2015 RCT	E = 31/5~6y/NC C = 30/5~6y/NC	8 weeks (2 x/week) moderate-intensity (60–70% HRmax) football games (O, S), 35 min/time (E) vs. blank control (C)	②Panda-Lion task ②Snow-Grass task ③Corsi blocks test ③Reverse Corsi blocks test ④Flexible Item Selection task	+ + 0 0 0
Schmidt et al. [51], 2015 RCT	E1 = 69/11.3 ± 0.6y/62.3% E2 = 57/11.3 ± 0.6y/50.9% C = 55/11.4 ± 0.6y/49.1%	6 weeks (2 x/week) high-intensity soft hockey and basketball games (O, S), 45 min/time (E1) vs. 200 m round-trip run (Cl, Co) (E2) vs. traditional PE course (C)	②Flanker (E1 > E2 = C) ③2-back (E1 > E2 = C) ④More-odd shifting (E1 > E2 = C)	+0& +0& +0&
Martín-Martínez et al. [71], 2015 non-RCCT	54/15~16y/25.9%	8 weeks (2 x/week) moderate-intensity (RPE = 13.36 ± 1.39) group ball (football, basketball, and handball) games (O, S), 30–60 min/time (E) vs. aerobic exercise and modern dance (C)	②Stroop ③WISC-IV-digital and letter Span ④Trail Making Test	+ + +
Gallotta et al. [52], 2015b RCT	E1 = 56/8~11y/NC E2 = 59/8~11y/NC C = 41/8~11y/NC	20 weeks (2 x/week) moderate-intensity (RPE = 5~8) traditional PE course focusing on cardiovascular fitness, 60 min/time (E1) vs. activities focusing on improving coordination and flexibility (E2) vs. blank control (C)	②d2-test (E2 > E1)	+&
Chen et al. [72], 2016a RCT	E = 20/11.4 ± 0.6y/NC C = 20/11.3 ± 06y/NC	8 weeks (3 x/week) moderate-intensity (60–69% HRmax) mind–body aerobics (Cl, S), 40 min/time (E) vs. regular academics (C)	②Flanker ③1-back ④More-odd shifting	0 + +
Koutsandreou et al. [53], 2016 RCT	E1 = 27/9.3 ± 0.6y/NC E2 = 23/9.4 ± 0.7y/NC C = 21/9.3 ± 0.6y/NC	10 weeks (3 x/week) moderate-intensity (60–70% HRmax) aerobic exercise, 45 min/time (E1) vs. moderate intensity (55–65% HRmax) skill practice focused on improving coordination (S) (E2) vs. doing their homework (C)	③Letter Digit Span (E2 > E1)	+&
Alesi et al. [38], 2016 non-RCCT	E = 24/8.8 ± 1.1y/0.0% C = 20/9.3 ± 0.9y/0.0%	24 weeks (2 x/week) football intervention (O, S), 75 min/time (E) vs. traditional PE course, 1 x/week, 60 min/time (C)	①Tower of London ③Forward Digit Span ③Backward Digit Span ③Corsi blocks test	+ 0 0 +
Pesce et al. [73], 2016 RCT	E = 232/5~10y/50.4% C = 228/5~10y/49.6%	24 weeks (1 x/week) moderate intensity (131.9 ± 17.4 bpm) skill games (O, S) focusing on motor coordination and cognitive engagement, 60 min/time (E) vs. traditional PE course (C)	②RNG-inhibition of mental routines ③RNG-working memory updating	+ 0
Robinson et al. [74], 2016 RCT	E = 68/52.4 ± 5.2 m/39.7% C = 45/51.6 ± 5.2 m/40.0%	5 weeks (3 x/week) Children’s Health Activity Programme (motor-skills-led intervention), 40 min/time (E) vs. outdoor free play (C)	②Delay of gratification snack task	+
van der Niet et al. [75], 2016 non-RCCT	E = 53/8.8 ± 0.8y/64.2% C = 52/8.9 ± 1.2y/38.5%	22 weeks (2 x/week) of moderate- to high-intensity running games, circuit training, and football with cognitive participation, 30 min/time (E) vs. blank control (C)	①Tower of London ②Stroop ③Visual Memory Span ③Digit Span ④Trailmaking test	0 + 0 + 0
Chen et al. [76], 2017 RCT	E = 21/9.4 ± 0.5y/47.6% C = 20/9.2 ± 0.4y/50.0%	8 weeks (2 x/week) moderate-intensity (60–69% HRmax) football intervention (O, S), 40 min/time (E) vs. traditional PE course (C)	②Flanker ③1-back ④More-odd shifting	+ + +
Cho et al. [77], 2017 RCT	E = 15/11.2 ± 0.8y/40.0% C = 15/11.3 ± 0.7y/40.0%	16 weeks (5 x/week) moderate intensity (RPE = 11~15) taekwondo intervention (O, S), 60 min/time (E) vs. blank control (C)	②Stroop	+
Xiong et al. [78], 2017 non-RCCT	39/4.67y/48.7%	12 weeks (7 x/week) structured motor-skills intervention, 30 min/time (E) vs. unstructured free play (C)	①WCST	+
Hsieh et al. [79], 2017 non-RCCT	E = 24/8.7 ± 1.1y/NC C = 20/8.6 ± 1.1y/NC	8 weeks (2 x/week) moderate-intensity (136.4 ± 16.8 bpm) gymnastics intervention (Cl, S), 90 min/time (E) vs. blank control (C)	③Delayed matching-to-sample test	+
Mulvey et al. [80], 2018 RCT	T = 50/3~6y/NC C = 57/3~6y/NC	6 weeks (2 x/week) SKIP program, 30 min/time (E) vs. rest as usual (C)	②HTKS	+
Lo et al. [81], 2019 BAS	E = NC/13.36 ± 1.15y/NC C = NC/13.47 ± 1.24y/NC	8 weeks judo (3 x/week) (O, S), 60 min/time (E) vs. never trained in judo (C)	④spatial task-switching	+
Dai et al. [82], 2020 non-RCCT	E = 46/10.5 ± 0.3y/NC C = 43/10.4 ± 0.3y/NC	24 weeks (5 x/week) moderate-intensity (60~69% HRmax) football intervention (O, S), 120 min/time (E) vs. blank control (C)	②Flanker ③2-back ④Salthouse	+ + +
Lai et al. [83], 2020 RCT	E = 10/5~7y/50.0% C = 10/5~7y/50.0%	8 weeks (2 x/week) moderate-intensity (60~69% HRmax) tennis intervention (O, S), 60 min/time (E) vs. basic academic course (C)	③1-back	+
Oppici et al. [54], 2020 RCT	E1 = 30/8.8 ± 0.5y/62.0% E2 = 30/8.7 ± 0.7y/59.0% C = 20/8.9 ± 0.7y/63.0%	7 weeks (2 x/week) high-cognitive dance practice (Cl, S), 60 min/time (E1) vs. low-cognitive dance practice (Cl, S) (E2) vs. blank control (C)	②Flanker ③List Sorting Working Memory test ④Dimensional Change Card Sort test	0 0 0
Chou et al. [84], 2020 RCT	E = 44/12.3 ± 0.7y/38.6% C = 40/12.1 ± 0.7y/37.5%	8 weeks (3 x/week) moderate- to high-intensity (60–80% HRmax) running games, jump-rope games, baseball, football, and basketball (O), 40 min/time (E) vs. traditional PE course (C)	②Stroop	+
Meijer et al. [55], 2021 RCT	E1 = 206/9.3 ± 0.7y/50.5% E2 = 235/9.0 ± 0.6y/53.6% C = 415/9.2 ± 0.7y/49.6%	14 weeks (4 x/week) aerobic-exercise intervention (Cl, Co), 30 min/time (E1) vs. team games with cognitive participation (O, S) (E2) vs. traditional PE sessions (C)	②Attention Network Test-interference control ②Stop Signal Task ③Digit Span ③Grid Task	0 0 0 0
Ma et al. [85], 2022 non-RCCT	E = 40/9.2 ± 0.3y/NC C = 40/9.2 ± 0.3y/NC	16 weeks (3 x/week) football intervention (O, S), 40 min/time (E) vs. blank control (C)	②GO/NO GO ③1-back ③2-back ④More-odd shifting	+ + + +

Abbreviations and notes: RCT: randomized controlled trial; RCD: randomized crossover design; non-RCCT: non-randomized concurrent control trial; BAS: before–after study; E: experimental group; C: control group; y: year; m: month; g: grade; F%: females as a percentage of subjects; O: open skills; Cl: closed skills; Co: continuous skills; S: sequential skills; NC: not clear; HRmax: maximum heart rate; RPE: rate of perceived exertion; ①: planning; ②; inhibitory control; ③: working memory; ④: cognitive flexibility; +: beneficial to experimental group; −: beneficial to control group; 0: no significant difference between the experimental and control groups; &: comparison of intervention results between experimental groups; Pre: pre-test; Post: post-test; PDTP: Picture Deletion Task for Preschoolers; DSST: Digit Symbol Substitution Test; RNG: Random Number Generation task; WCST: Wisconsin Card Sorting Test; WISC-IV: Wechsler Intelligence Scale for Children-Fourth Edition; HTKS: Head Toe Knee Shoulder test.

**Table 2:** Results of the quality assessment of the included RCTs and RCDs.

Included Articles	Randomization Methods	Blinding	Allocation Concealment	Completeness of Outcome Data	Selective Reporting of Study Results	Other Biases
Budde et al. [56], 2008	NC	NC	NC	Complete	No	NC
Niemann et al. [57], 2013	NC	Subject blinding	NC	Complete	No	NC
Palmer et al. [58], 2013	NC	NC	NC	Complete	No	NC
Yan et al. [40], 2014	NC	Subject blinding	NC	Complete	No	NC
Chen et al. [41], 2014a	Cluster randomization	NC	NC	Complete	No	NC
Chen et al. [59], 2014b	Stratified randomization	NC	NC	5 lost to follow-up	No	NC
Chen et al. [42], 2015a	Cluster randomization	NC	NC	Complete	No	NC
Chen et al. [60], 2015b	NC	NC	NC	Complete	No	NC
Jäger et al. [43], 2015	NC	Assessor blinding	NC	Complete	No	NC
Gallotta et al. [44], 2015a	Cluster randomization	NC	NC	Complete	No	NC
Cooper et al. [61], 2016	NC	Subject blinding	NC	Complete	No	NC
Stein et al. [62], 2017	NC	NC	NC	Complete	No	NC
O’Brien et al. [37], 2021	NC	NC	NC	5 lost to follow-up	No	NC
Ottoboni et al. [45], 2021	Stratified randomization	NC	NC	Complete	No	NC
Manjunath et al. [39], 2001	Random number table	NC	NC	Complete	No	NC
Lakes et al. [63], 2004	NC	Assessor blinding	NC	12 lost to follow-up	No	NC
Davis et al. [46], 2011	Stratified randomization	Double blinding	NC	Complete	No	NC
Kamijo et al. [64], 2011	NC	NC	NC	7 lost to follow-up	No	NC
Fisher et al. [65], 2011	Cluster randomization	Subject blinding	NC	8 lost to follow-up	No	NC
Lakes et al. [66], 2013	NC	Assessor blinding	NC	Complete	No	NC
Telles et al. [48], 2013	Cmputer-generated random numbers	Assessor blinding	paper hide	Complete	No	NC
Crova et al. [67], 2014	Cluster randomization	NC	NC	Complete	No	NC
Yin et al. [49], 2014	NC	NC	NC	Complete	No	NC
Hillman et al. [68], 2014	NC	Subject blinding	NC	15 lost to fllow-up	No	NC
Krafft et al. [69], 2014	NC	NC	NC	12 lost to follow-up	No	NC
Yin et al. [50], 2015	Stratified randomization	NC	NC	Complete	No	NC
Jiang et al. [70], 2015	NC	NC	NC	Complete	No	NC
Schmidt et al. [51], 2015	NC	Assessor blinding	NC	8.6% lost to follow-up	No	NC
Gallotta et al. [52], 2015b	Cluster randomization	NC	NC	Complete	No	NC
Chen et al. [72], 2016a	Drawing lots	Double blinding	NC	Complete	No	NC
Koutsandreou et al. [53], 2016	NC	Assessor blinding	NC	28 lost to follow-up	No	NC
Pesce et al. [73], 2016	Stratified randomization	NC	NC	17 lost to follow-up	No	NC
Robinson et al. [74], 2016	NC	NC	NC	42.5% lost to follow-up	No	NC
Chen et al. [76], 2017	Drawing lots	Double blinding	NC	7 lost to follow-up	No	NC
Cho et al. [77], 2017	NC	NC	NC	Complete	No	NC
Mulvey et al. [80], 2018	Random number table	NC	NC	Complete	No	NC
Lai et al. [83], 2020	NC	Double blinding	NC	12 lost to follow-up	No	NC
Oppici et al. [54], 2020	Stratified randomization	Subject blinding	NC	2 lost to follow-up	No	NC
Chou et al. [84], 2020	NC	NC	NC	Complete	No	NC
Meijer et al. [55], 2021	Cluster randomization	No	NC	35 lost to follw-up	No	NC

Abbreviations and notes: NC: not clear.

**Table 3:** Results of the quality assessment of the included non-RRCTs and BAS.

Included Articles	(1)	(2)	(3)	(4)	(5)	(6)	(7)	(8)	(9)	(10)	(11)	(12)	Total
Chang et al. [47], 2013	2	2	2	2	0	2	2	0	2	2	1	2	19
Martín-Martínez et al. [71], 2015	2	2	2	2	0	2	2	0	2	2	2	2	20
Alesi et al. [38], 2016	2	1	2	2	0	2	2	0	2	2	2	2	19
van der Niet et al. [75], 2016	2	2	2	2	0	2	0	0	2	2	1	2	17
Xiong et al. [78], 2017	2	1	2	2	0	2	0	0	2	2	1	2	16
Hsieh et al. [79], 2017	2	2	2	2	2	2	2	2	2	2	2	2	24
Lo et al. [81], 2019	2	2	2	2	0	2	2	1	NA	NA	NA	NA	13
Dai et al. [82], 2020	2	1	2	2	0	2	0	0	2	2	2	2	17
Ma et al. [85], 2022	2	0	2	2	0	2	1	0	2	2	2	2	17

Abbreviations and notes: (1) the study purpose is clearly given; (2) consistency of the included subjects; (3) collection of expected data; (4) the outcome indicators can properly reflect the study purpose; (5) objectivity of outcome index evaluation; (6) whether the follow-up time is sufficient; (7) the rate of lost visits is lower than 5%; (8) whether the sample size has been estimated; (9) whether the control group has been properly selected; (10) whether the control group is synchronized; (11) whether the baseline between groups is comparable; (12) whether the statistical analysis is appropriate; NA: not applicable.

**Table 4:** Summary of intergroup comparisons of acute-intervention effectiveness.

Characteristic	Inhibitory Control		Working Memory		Cognitive Flexibility	
	Effective Rate (%)	*χ* ^2^	Effective Rate (%)	*χ* ^2^	Effective Rate (%)	*χ* ^2^
Total	14/18 (77.8)		12/16 (75.0)		7/13 (53.8)	
**Age**						
Pre-school	1/25 (0.0)	1.004	—		0/1 (0.0)	1.264
Post-preschool	13/16 (81.3)		11/15 (73.3)		7/12 (58.3)	
**Intensity**						
Low	1/1 (100.0)	1.231	0/1 (0.0)	2.703	0/1 (0.0)	5.600
Moderate	9/12 (77.0)		7/9 (77.7)		7/9 (77.8)	
High	3/3 (100.0)		3/4 (75.0)		0/2 (0.0)	
**Duration**						
10~20 min	3/5 (60.0)	2.714	0/2 (0.0)	6.857 **	0/3 (0.0)	4.550 *
30~50 min	12/13 (92.3)		12/14 (85.7)		7/10 (70.0)	
**Skill type**						
Open skills	3/6 (50.0)	4.018 *	5/6 (83.3)	0.356	2/3 (66.7)	0.258
Closed skills	11/12 (91.7)		7/10 (70.0)		5/10 (50.0)	
Contionous skills	7/8 (87.5)	0.788	6/8 (75.0)	0.000	4/6 (66.7)	0.343
Sequential skills	7/10 (70.0)		6/8 (75.0)		3/6 (50.0)	
Open–Continous	2/2 (100.0)	8.839 *	3/3 (100.0)	1.778	2/2 (100.0)	2.940
Open–Sequential	1/4 (25.0)		2/3 (66.7)		0/1 (0.0%)	
Closed–Contionous	5/6 (83.3)		3/5 (60.0)		2/4 (50.0)	
Closed–Sequential	6/6 (100.0)		4/5 (80.0)		3/6 (50.0)	

Abbreviations and notes: For the measurement of the same dimension using multiple tools, as long as one positive benefit is achieved, it will be included in the effective intervention; * *p* < 0.05; ** *p* < 0.01.

**Table 5:** Summary of intergroup comparisons of long-term intervention effectiveness.

Characteristic	Planning		Inhibitory Control		Working Memory		Cognitive Flexibility	
	Effective Rate (%)	*χ* ^2^	Effective Rate (%)	*χ* ^2^	Effective Rate (%)	*χ* ^2^	Effective Rate (%)	*χ* ^2^
Total	4/7 (57.1)		25/36 (69.4)		19/31 (61.3)		12/20 (60.0)	
**Age**								
Pre-school	1/2 (50.0)	0.058	4/5 (80.0)	0.354	2/3 (66.7)	0.002	0/1 (0.0)	1.579
Post-preschool	3/5 (60.0)		20/30 (66.7)		17/26 (65.4)		12/19 (63.2)	
**Intensity**								
Low	—		1/1 (100.0)	1.920	—	0.762	—	1.148
Moderate	1/2 (50.0)		13/15 (86.7)		11/14 (78.6)		6/7 (85.7)	
High	—		1/2 (50.0)		1/2 (50.0)		1/2 (50.0)	
**Period**								
≤8 weeks	1/1 (100.0)	0.875	8/12 (66.7)	1.833	5/9 (55.6)	2.245	5/9 (55.6)	0.185
9~16 weeks	2/4 (50.0)		7/12 (58.3)		6/12 (50.0)		4/6 (66.7)	
≥17 weeks	1/2 (50.0)		10/12 (83.3)		8/10 (80.0)		3/5 (60.0)	
**Frequency**								
<3 x/week	1/1 (100.0)	0.600	8/12 (66.7)	0.203	5/12 (41.7)	2.801	3/8 (37.5)	2.036
≥3 x/week	3/5 (60.0)		17/23 (73.9)		13/18 (72.2)		9/12 (75.0)	
**Duration**								
≤30 min	1/3 (33.3)	1.556	6/10 (60.0)	1.190	5/10 (50.0)	0.883	5/8 (62.5)	0.173
30~50 min	1/1 (100.0)		12/15 (80.0)		7/10 (70.0)		4/7 (57.1)	
>50 min	2/3 (66.7)		7/10 (70.0)		7/11 (63.6)		2/4 (50.0)	
**Skill type**								
Open skills	1/1 (100.0)	0.000	13/16 (81.3)	5.740 *	9/14 (64.3)	1.473	8/10 (80.0)	3.484
Closed skills	1/1 (100.0)		3/9 (33.3)		3/8 (37.5)		2/6 (33.3)	
Contionous skills	—		3/7 (42.9)	1.627	3/6 (50.0)	0.403	3/5 (60.0)	0.019
Sequential skills	2/2 (100.0)		12/17 (70.6)		11/17 (64.7)		7/11 (63.6)	
Open–Continous		0.000	2/3 (66.7)	9.555 *	1/3 (33.3)	3.311	2/3 (66.7)	5.526
Open–Sequential	1/1 (100.0)		11/12 (91.7)		7/11 (63.6)		6/7 (85.7)	
Closed–Contionous			1/3 (33.3)		0/2 (0.0)		0/1 (0.0)	
Closed–Sequential	1/1 (100.0)		1/5 (20.0)		2/5 (40.0)		1/4 (25.0)	

Abbreviations and notes: for the measurement of the same dimension using multiple tools, as long as one positive benefit is achieved, it will be included in the effective intervention; * *p* < 0.05.

## Data Availability

No data were used to support this study.

## References

[1] Friedman N.P., Robbins T.W. (2022). The role of prefrontal cortex in cognitive control and executive function. Neuropsychopharmacology.

[2] Ferguson H.J., Brunsdon V.E.A., Bradford E.E.F. (2021). The developmental trajectories of executive function from adolescence to old age. Sci. Rep..

[3] Baddeley A. (1996). Exploring the central executive. Q. J. Exp. Psychol. Sect. A.

[4] Hofmann W., Schmeichel B.J., Baddeley A.D. (2012). Executive functions and self-regulation. Trends Cogn. Sci..

[5] Baggetta P., Alexander P.A. (2016). Conceptualization and operationalization of executive function. Mind Brain Educ..

[6] Montoya M.F., Susperreguy M.I., Dinarte L., Morrison F.J., San Martin E., Rojas-Barahona C.A., Förster C.E. (2019). Executive function in Chilean preschool children: Do short-term memory, working memory, and response inhibition contribute differentially to early academic skills?. Early Child. Res. Q..

[7] Allan J.L., McMinn D., Daly M. (2016). A bidirectional relationship between executive function and health behavior: Evidence, implications, and future directions. Front. Neurosci..

[8] Liu C., Dai J., Chen Y., Qi Z., Xin F., Zhuang Q., Zhou X., Zhou F., Luo L., Huang Y. (2021). Disorder-and emotional context-specific neurofunctional alterations during inhibitory control in generalized anxiety and major depressive disorder. Neuro Image Clin..

[9] Thomas A.G., Dennis A., Bandettini P.A., Johansen-Berg H. (2012). The effects of aerobic activity on brain structure. Front. Psychol..

[10] Berchtold N.C., Castello N., Cotman C.W. (2010). Exercise and time-dependent benefits to learning and memory. Neuroscience.

[11] Hillman C.H., Logan N.E., Shigeta T.T. (2019). A review of acute physical activity effects on brain and cognition in children. Transl. J. Am. Coll. Sport. Med..

[12] Weinstein A.M., Voss M.W., Prakash R.S., Chaddock L., Szabo A., White S.M., Wojcicki T., Mailey E., McAuley E., Kramer A.F. (2012). The association between aerobic fitness and executive function is mediated by prefrontal cortex volume. Brain Behav. Immun..

[13] Xiong X., Zhu L.N., Dong X.X., Wang W., Yan J., Chen A.G. (2018). Aerobic exercise intervention alters executive function and white matter integrity in deaf children: A randomized controlled study. Neural Plast..

[14] Chaddock-Heyman L., Erickson K.I., Voss M.W., Knecht A.M., Pontifex M.B., Castelli D.M., Charles H.H., Kramer A.F. (2013). The effects of physical activity on functional MRI activation associated with cognitive control in children: A randomized controlled intervention. Front. Hum. Neurosci..

[15] Meijer A., Königs M., Pouwels P.J., Smith J., Visscher C., Bosker R.J., Hartman E., Oosterlaan J. (2022). Effects of aerobic versus cognitively demanding exercise interventions on brain structure and function in healthy children—Results from a cluster randomized controlled trial. Psychophysiology.

[16] Ludyga S., Gerber M., Kamijo K. (2022). Exercise types and working memory components during development. Trends Cogn. Sci..

[17] Chai J., Lin J.B. (2018). Changing Law of Primary and Secondary School Students’ Interest in Sports Events. J. Shenyang Sport Univ..

[18] Zhao X., Chen L., Fu L., Maes J.H. (2015). “Wesley says”: A children’s response inhibition playground training game yields preliminary evidence of transfer effects. Front. Psychol..

[19] Vazou S., Pesce C., Lakes K., Smiley-Oyen A. (2019). More than one road leads to Rome: A narrative review and meta-analysis of physical activity intervention effects on cognition in youth. Int. J. Sport Exerc. Psychol..

[20] Pesce C., Croce R., Ben-Soussan T.D., Vazou S., McCullick B., Tomporowski P.D., Horvat M. (2019). Variability of practice as an interface between motor and cognitive development. Int. J. Sport Exerc. Psychol..

[21] Diamond A., Ling D.S. (2016). Conclusions about interventions, programs, and approaches for improving executive functions that appear justified and those that, despite much hype, do not. Dev. Cogn. Neurosci..

[22] Zhang Y.B. (2012). Athletic Skill Study Theory and Practice.

[23] Zhu H., Chen A., Guo W., Zhu F., Wang B. (2020). Which type of exercise is more beneficial for cognitive function? A meta-analysis of the effects of open-skill exercise versus closed-skill exercise among children, adults, and elderly populations. Appl. Sci..

[24] Tomporowski P.D., Pesce C. (2019). Exercise, sports, and performance arts benefit cognition via a common process. Psychol. Bull..

[25] Sale A., Berardi N., Maffei L. (2009). Enrich the environment to empower the brain. Trends Neurosci..

[26] Chueh T.Y., Huang C.J., Hsieh S.S., Chen K.F., Chang Y.K., Hung T.M. (2017). Sports training enhances visuo-spatial cognition regardless of open-closed typology. PeerJ.

[27] Becker D.R., McClelland M.M., Geldhof G.J., Gunter K.B., MacDonald M. (2018). Open-skilled sport, sport intensity, executive function, and academic achievement in grade school children. Early Educ. Dev..

[28] Wheat J.S., Glazier P.S. (2006). Chapter 9—Measuring coordination and variability in coordination. Movement System Variability.

[29] Rigoli D., Piek J.P., Kane R., Oosterlaan J. (2012). An examination of the relationship between motor coordination and executive functions in adolescents. Dev. Med. Child Neurol..

[30] Michel E., Molitor S., Schneider W. (2019). Motor coordination and executive functions as early predictors of reading and spelling acquisition. Dev. Neuropsychol..

[31] Bernardi M., Leonard H.C., Hill E.L., Botting N., Henry L.A. (2018). Executive functions in children with developmental coordination disorder: A 2-year follow-up study. Dev. Med. Child Neurol..

[32] Schmidt R.A., Wrisberg C.A. (2008). Motor Learning and Performance: A Situation-Based Learning Approach.

[33] Voss M.W., Kramer A.F., Basak C., Prakash R.S., Roberts B. (2010). Are expert athletes ‘expert’in the cognitive laboratory? A meta-analytic review of cognition and sport expertise. Appl. Cogn. Psychol..

[34] Costantino G., Montano N., Casazza G. (2015). When should we change our clinical practice based on the results of a clinical study? Searching for evidence: PICOS and PubMed. Intern. Emerg. Med..

[35] Cumpston M., Li T., Page M.J., Chandler J., Welch V.A., Higgins J.P., Thomas J. (2019). Updated guidance for trusted systematic reviews: A new edition of the Cochrane Handbook for Systematic Reviews of Interventions. Cochrane Database Syst. Rev..

[36] Slim K., Nini E., Forestier D., Kwiatkowski F., Panis Y., Chipponi J. (2003). Methodological index for non-randomized studies (MINORS): Development and validation of a new instrument. ANZ J. Surg..

[37] O’Brien J., Ottoboni G., Tessari A., Setti A. (2021). Multisensory Perception, Verbal, Visuo-spatial and motor working memory modulation after a single open-or closed-skill exercise session in children. J. Cogn. Enhanc..

[38] Alesi M., Bianco A., Luppina G., Palma A., Pepi A. (2016). Improving children’s coordinative skills and executive functions: The effects of a football exercise program. Percept. Mot. Ski..

[39] Manjunath N.K., Telles S. (2001). Improved performance in the Tower of London test following yoga. Indian J. Physiol. Pharmacol..

[40] Yan J., Wang Y., Chen A.G., Ma D.J. (2014). Empirical study of the impact of various school-term physical activity of moderate intensity on the executive function of children in their preadolescence. J. Sport. Sci..

[41] Chen A.G., Zhao L., Li H.Y., Yan J., Yin H.C. (2014). Effects of acute basketball dribbling training of different intensity on executive function of primary students. J. TUS.

[42] Chen A.G., Zhao Z.Y., Yan J. (2015). Effects of rope skipping with different forms of organization on the executive function of preadolescent children: A school-based experimental study. Chin. J. Sport. Med..

[43] Jäger K., Schmidt M., Conzelmann A., Roebers C.M. (2015). The effects of qualitatively different acute physical activity interventions in real-world settings on executive functions in preadolescent children. Ment. Health Phys. Act..

[44] Gallotta M.C., Emerenziani G.P., Franciosi E., Meucci M., Guidetti L., Baldari C. (2015). Acute physical activity and delayed attention in primary school students. Scand. J. Med. Sci. Sport..

[45] Ottoboni G., Ceciliani A., Tessari A. (2021). The effect of structured exercise on short-term memory subsystems: New insight on training activities. Int. J. Environ. Res. Public Health.

[46] Davis C.L., Tomporowski P.D., McDowell J.E., Austin B.P., Miller P.H., Yanasak N.E., Allison J.D., Naglieri J.A. (2011). Exercise improves executive function and achievement and alters brain activation in overweight children: A randomized, controlled trial. Health Psychol..

[47] Chang Y.K., Tsai Y.J., Chen T.T., Hung T.M. (2013). The impacts of coordinative exercise on executive function in kindergarten children: An ERP study. Exp. Brain Res..

[48] Telles S., Singh N., Bhardwaj A.K., Kumar A., Balkrishna A. (2013). Effect of yoga or physical exercise on physical, cognitive and emotional measures in children: A randomized controlled trial. Child Adolesc. Psychiatry Ment. Health.

[49] Yin H.C., Chen A.G., Ma Z., Li X.N., Liu M. (2014). A follow-up study on two kinds of exercise intervention programs for children’s executive functions. China Sport Sci..

[50] Yin H.C., Li X.N., Chen A.G., Song X.Q., Du Y., Pan J.L., Wang C. (2015). Experimental study on the effects of five kinds of exercise intervention programs on brain executive functions of primary students. J. TUS.

[51] Schmidt M., Jäger K., Egger F., Roebers C.M., Conzelmann A. (2015). Cognitively engaging chronic physical activity, but not aerobic exercise, affects executive functions in primary school children: A group-randomized controlled trial. J. Sport Exerc. Psychol..

[52] Gallotta M.C., Emerenziani G.P., Iazzoni S., Meucci M., Baldari C., Guidetti L. (2015). Impacts of coordinative training on normal weight and overweight/obese children’s attentional performance. Front. Hum. Neurosci..

[53] Koutsandreou F., Wegner M., Niemann C., Budde H. (2016). Effects of Motor versus Cardiovascular Exercise Training on Children’s Working Memory. Med. Sci. Sport. Exerc..

[54] Oppici L., Rudd J.R., Buszard T., Spittle S. (2020). Efficacy of a 7-week dance (RCT) PE curriculum with different teaching pedagogies and levels of cognitive challenge to improve working memory capacity and motor competence in 8–10 years old children. Psychol. Sport Exerc..

[55] Meijer A., Königs M., van der Fels I.M., Visscher C., Bosker R.J., Hartman E., Oosterlaan J. (2020). The effects of aerobic versus cognitively demanding exercise interventions on executive functioning in school-aged children: A cluster-randomized controlled trial. J. Sport Exerc. Psychol..

[56] Budde H., Voelcker-Rehage C., Pietraßyk-Kendziorra S., Ribeiro P., Tidow G. (2008). Acute coordinative exercise improves attentional performance in adolescents. Neurosci. Lett..

[57] Niemann C., Wegner M., Voelcker-Rehage C., Holzweg M., Arafat A.M., Budde H. (2013). Influence of acute and chronic physical activity on cognitive performance and saliva testosterone in preadolescent school children. Ment. Health Phys. Act..

[58] Palmer K.K., Miller M.W., Robinson L.E. (2013). Acute exercise enhances preschoolers’ ability to sustain attention. J. Sport. Exerc. Psychol..

[59] Chen A.G., Yan J., Yin H.C., Pan C.Y., Chang Y.K. (2014). Effects of acute aerobic exercise on multiple aspects of executive function in preadolescent children. Psychol. Sport Exerc..

[60] Chen A.G., Feng L., Zhu L.N., Yan J. (2015). Effect of moderate intensity basketball dribbling teaining of different durations on children’ executive function. J. Cap. Univ. Phys. Educ. Sport..

[61] Cooper S.B., Bandelow S., Nute M.L., Dring K.J., Stannard R.L., Morris J.G., Nevill M.E. (2016). Sprint-based exercise and cognitive function in adolescents. Prev. Med. Rep..

[62] Stein M., Auerswald M., Ebersbach M. (2017). Relationships between motor and executive functions and the effect of an acute coordinative intervention on executive functions in kindergartners. Front. Psychol..

[63] Lakes K.D., Hoyt W.T. (2004). Promoting self-regulation through school-based martial arts training. J. Appl. Dev. Psychol..

[64] Kamijo K., Pontifex M.B., O’Leary K.C., Scudder M.R., Wu C.T., Castelli D.M., Hillman C.H. (2011). The effects of an afterschool physical activity program on working memory in preadolescent children. Dev. Sci..

[65] Fisher A., Boyle J.M., Paton J.Y., Tomporowski P., Watson C., McColl J.H., Reilly J.J. (2011). Effects of a physical education intervention on cognitive function in young children: Randomized controlled pilot study. BMC Pediatr..

[66] Lakes K.D., Bryars T., Sirisinahal S., Salim N., Arastoo S., Emmerson N., Kang D., Shim L., Wong D., Kang C.J. (2013). The healthy for life taekwondo pilot study: A preliminary evaluation of effects on executive function and BMI, feasibility, and acceptability. Ment. Health Phys. Act..

[67] Crova C., Struzzolino I., Marchetti R., Masci I., Vannozzi G., Forte R., Pesce C. (2014). Cognitively challenging physical activity benefits executive function in overweight children. J. Sport. Sci..

[68] Hillman C.H., Pontifex M.B., Castelli D.M., Khan N.A., Raine L.B., Scudder M.R., Drolette E.S., Moore R.D., Wu C.T., Kamijo K. (2014). Effects of the FITKids randomized controlled trial on executive control and brain function. Pediatrics.

[69] Krafft C.E., Schwarz N.F., Chi L., Weinberger A.L., Schaeffer D.J., Pierce J.E., Rodrigue A.L., Yanasak N.E., Miller P.H., Tomporowski P.D. (2014). An 8-month randomized controlled exercise trial alters brain activation during cognitive tasks in overweight children. Obesity.

[70] Jiang D.L., Zeng C.Z. (2015). The effect of 8-week soccer exercise with moderate intensity on executive function in preschool children. China Sport Sci. Technol..

[71] Martín-Martínez I., Chirosa-Ríos L.J., Reigal-Garrido R.E., Hernández-Mendo A., Juárez-Ruiz-de-Mier R., Guisado-Barrilao R. (2015). Efectos de la actividad física sobre las funciones ejecutivas en una muestra de adolescentes. An. Psicol..

[72] Chen A.G., Liang H.Y., Yan J., Yin H.C. (2016). The developmental features of executive functions of left-at-home children and the interventions by mind-body exercise. Chin. J. Spec. Educ..

[73] Pesce C., Masci I., Marchetti R., Vazou S., Sääkslahti A., Tomporowski P.D. (2016). Deliberate play and preparation jointly benefit motor and cognitive development: Mediated and moderated effects. Front. Psychol..

[74] Robinson L.E., Palmer K.K., Bub K.L. (2016). Effect of the children’s health activity motor program on motor skills and self-regulation in head start preschoolers:an efficacy trial. Front. Public Health.

[75] Van der Niet A.G., Smith J., Oosterlaan J., Scherder E.J., Hartman E., Visscher C. (2016). Effects of a cognitively demanding aerobic intervention during recess on children’s physical fitness and executive functioning. Pediatr. Exerc. Sci..

[76] Chen A.G., Chen L.P., Yan J. (2017). Exeperimental study on the effect of eight-week football program on executive function among left-behind children. J. Shandong Sport Univ..

[77] Cho S.Y., So W.Y., Roh H.T. (2017). The effects of taekwondo training on peripheral neuroplasticity-related growth factors, cerebral blood flow velocity, and cognitive functions in healthy children: A randomized controlled trial. Int. J. Environ. Res. Public Health.

[78] Xiong S., Li X., Tao K. (2017). Effects of structured physical activity program on Chinese young children’s executive functions and perceived physical competence in a day care center. BioMed Res. Int..

[79] Hsieh S.S., Lin C.C., Chang Y.K., Huang C.J., Hung T.M., City T. (2017). Effects of childhood gymnastics program on spatial working memory. Med. Sci. Sport. Exerc..

[80] Mulvey K.L., Taunton S., Pennell A., Brian A. (2018). Head, toes, knees, SKIP! Improving preschool children’s executive function through a motor competence intervention. J. Sport Exerc. Psychol..

[81] Lo W.L.A., Liang Z., Li W., Luo S., Zou Z., Chen S., Yu Q. (2019). The effect of judo training on set-shifting in school children. BioMed Res. Int..

[82] Dai C. (2020). Effects of soccer exercise and stop practice on executive function of school age children. J. Chengdu Sport Univ..

[83] Lai Y., Wang Z., Yue G.H., Jiang C. (2020). Determining whether tennis benefits the updating function in young children: A functional near-Infrared spectroscopy study. Appl. Sci..

[84] Chou C.C., Chen K.C., Huang M.Y., Tu H.Y., Huang C.J. (2020). Can movement games enhance executive function in overweight children? A randomized controlled trial. J. Teach. Phys. Educ..

[85] Ma Y.D., Xu L., Fu Q. (2022). An empirical study of the impact of campus football training on children’s mental health. J. Shenyang Sport Univ..

[86] Pennington B.F., Ozonoff S. (1996). Executive functions and developmental psychopathology. J. Child Psychol. Psychiatry.

[87] Miyake A., Friedman N.P., Emerson M.J., Witzki A.H., Howerter A., Wager T.D. (2000). The unity and diversity of executive functions and their contributions to complex “frontal lobe” tasks: A latent variable analysis. Cogn. Psychol..

[88] Smith E.E., Jonides J. (1999). Storage and executive processes in the frontal lobes. Science.

[89] Diamond A. (2013). Executive functions. Annu. Rev. Psychol..

[90] Anderson P., Anderson V., Garth J. (2001). Assessment and development of organizational ability: The Rey complex figure organizational strategy score (RCF-OSS). Clin. Neuropsychol..

[91] Chang Y.K., Tsai C.L., Hung T.M., So E.C., Chen F.T., Etnier J.L. (2011). Effects of acute exercise on executive function: A study with a Tower of London Task. J. Sport Exerc. Psychol..

[92] Anderson V., Northam E., Wrennall J. (2018). Developmental Neuropsychology: A Clinical Approach.

[93] Wang J.F., Qi C.Z., Wei X.N. (2019). Effects of physical activity on executive function: A meta-analysis. J. Cap. Univ. Phys. Educ. Sport..

[94] Chen A.G., Yin H.C., Yan J., Yang Y. (2011). Effects of acute aerobic exercise of different intensity on executive function. Acta Psychol. Sin..

[95] Baumeister R.F., Vohs K.D., Tice D.M. (2007). The strength model of self-control. Curr. Dir. Psychol. Sci..

[96] Larson E.B., Wang L.I., Bowen J.D., McCormick W.C., Teri L., Crane P., Kukull W. (2006). Exercise is associated with reduced risk for incident dementia among persons 65 years of age and older. Ann. Intern. Med..

[97] De Souto Barreto P., Delrieu J., Andrieu S., Vellas B., Rolland Y. (2016). Physical activity and cognitive function in middle-aged and older adults: An analysis of 104,909 people from 20 countries. Mayo Clin. Proc..

[98] Chang Y.K., Chu C.H., Wang C.C., Wang Y.C., Song T.F., Tsai C.L., Etnier J.L. (2015). Dose-response relation between exercise duration and cognition. Med. Sci. Sports Exerc..

[99] Audiffren M., André N. (2015). The strength model of self-control revisited: Linking acute and chronic effects of exercise on executive functions. J. Sport Health Sci..

[100] Voelcker-Rehage C., Godde B., Staudinger U.M. (2011). Cardiovascular and coordination training differentially improve cognitive performance and neural processing in older adults. Front. Hum. Neurosci..

[101] Teitelbaum P., Teitelbaum O. (2007). FMRI study: Visuospatial processing and the function of the prefrontal-parietal network in autism-type spectrum disorders. Clin. Med. Eng..

[102] Mavilidi M.F., Okely A.D., Chandler P., Cliff D.P., Paas F. (2015). Effects of integrated physical exercises and gestures on preschool children’s foreign language vocabulary learning. Educ. Psychol. Rev..

[103] Moreau D., Morrison A.B., Conway A.R.A. (2015). An ecological approach to cognitive enhancement: Complex motor training. Acta Psychol..

[104] Carey J.R., Bhatt E., Nagpal A. (2005). Neuroplasticity promoted by task complexity. Exerc. Sport Sci. Rev..

[105] Diamond A. (2015). Effects of physical exercise on executive functions: Going beyond simply moving to moving with thought. Ann. Sport. Med. Res..

[106] Krafft C.E., Schaeffer D.J., Schwarz N.F., Chi L., Weinberger A.L., Pierce J.E., Rodrigue A.L., Allison J.D., Yanasak N.E., Liu T. (2014). Improved frontoparietal white matter integrity in overweight children is associated with attendance at an after-school exercise program. Dev. Neurosci..

